# Motivational goal-priming with or without awareness produces faster and stronger force exertion

**DOI:** 10.1038/s41598-018-28410-0

**Published:** 2018-07-04

**Authors:** Yudai Takarada, Daichi Nozaki

**Affiliations:** 1 Faculty of Sports Sciences, Waseda University, 2-579-15 Tokorozawa, Saitama, 359-1192 Japan; 2 Graduate School of Education, The University of Tokyo, Bunkyo-ku, Tokyo 113-0033 Japan

## Abstract

Previous research has demonstrated that barely visible (subliminal) goal-priming with motivational reward can alter the state of the motor system and enhance motor output. Research shows that these affective-motivational effects result from associations between goal representations and positive affect without conscious awareness. Here, we tested whether motivational priming can increase motor output even if the priming is fully visible (supraliminal), and whether the priming effect occurs through increased cortical excitability. Groups of participants were primed with either barely visible or fully visible words related to effort and control sequences of random letters that were each followed by fully visible positively reinforcing words. The priming effect was measured behaviourally by handgrip force and reaction time to the grip cue after the priming was complete. Physiologically, the effects were measured by pupil dilation and motor-evoked potentials (MEPs) in response to transcranial magnetic stimulation during the priming task. Analysis showed that for both the supraliminal and subliminal conditions, reaction time decreased and total force, MEP magnitude, and pupil dilation increased. None of the priming-induced changes in behaviour or physiology differed significantly between the supraliminal and the subliminal groups, indicating that implicit motivation towards motor goals might not require conscious perception of the goals.

## Introduction

Decisions about motor actions are completed before we are aware of having made them. Indeed, Libet *et al*.^1^ showed that readiness potential—a change in electroencephalographic (EEG) activity over the motor cortex—begins more than 350 ms before a person becomes aware of the decision to act. Moreover, the activity in several cortical regions, such as the precuneus and the fronto-polar cortex, reflects decision classification up to 2–3 seconds before an individual becomes aware of their decision to act. Thereafter, the supplementary motor area (SMA) determines the timing of that decision^2^. These studies indicate that voluntary action is initiated unconsciously, with movement selection always preceding awareness, and the feeling of intention (sense of self-agency) or sense of ownership coming afterward. This scenario also fits with Hallet’s model that treats free will as a perception^3^.

Focussing on the automatic and unconscious motor activation processes, we have shown that subliminal goal-priming with motivational reward actually altered the background state of the motor system, resulting in a 7% increase in the force level of the maximum voluntary hand-grip contraction^4^. This kind of affective-motivational effect on the motor system is related to both the reward-associated dopaminergic system and the pupil-linked neuromodulatory system^5^, which have functionally and anatomically close connections^6^. The interaction between these systems and between them and their mutual connections to the PFC is regulated by the locus ceruleus (LC)^6^. The release of noradrenaline from the LC leads to pupil dilation via α2-receptors, and thus dilation has been used extensively as an indirect measure of LC activity^7–9^. A large number of studies have reported that pupillary dilation is related to mental effort (cognitive load), and the correspondence between cognitive load and pupillary dilation has been documented in several contexts, including paired-associate learning^10,11^ and imagery tasks with abstract and concrete words^12–14^. The pupillary size at any time during a cognitive task reflects the subject’s participation in the task, and the precision of these changes has been identified to enable the second-by-second analysis of task-load and mental effort^15^. In humans, changes in pupil size at constant luminance have long been used as a marker of central autonomic processes linked to cognition, including attentional effort^15–17^.

Our decision to use a subliminal presentation of a behavioural (motor) goal in our studies was based on a previous study^18^. This procedure eliminates the possibility of demand-like properties, such as desirability of the goal, because participants cannot consciously detect the presented behavioural states during the affective shaping procedure. Thus, participants are unaware of the source for the association with positive affect. Positive affect can work as a reward signal that motivates behaviour^19,20^, and such reward signals can also motivate behaviour outside awareness^21,22^. Thus we consider those affective-motivational effects to result from associations between motor goal representations and positive affect without conscious awareness, which directly boosts physical resources. The current study substantially follows the earlier study with the same paradigm of the affective-motivational priming and conceptual analysis as those in the present study, in which physical exerting verbs have been shown to acts as motor goals^23^.

This line of thought is consistent with the previously proposed mechanism for unconscious goal pursuit. Unconscious goal pursuit is thought to be produced by two processes^24^. In the first, action is prepared at the perceptual, sensory, and motor levels, based on an ideomotor principle. In the second, positive reward signals are detected and processed in subcortical regions such as the basal ganglia in the limbic system, allowing effort for action to be recruited^21^. Coactivation of these processes may play a key role in the proposed mechanism of unconscious goal pursuit^4,23^.

Activating behavioural representations during action-word recognition potentiates the corresponding motor programs in the premotor cortex (PMC)^25^. One fMRI study showed that viewing action-related pictures, as well as mere mental imagery of actions (mirror system), activates the PMC in a somatotopic fashion^26^. Thus, compared with subliminal presentation, supraliminal presentation of the behavioural goal in reward goal-priming might cause greater enhancement of the affective-motivational effect on the motor system, including the PMC. Alternatively, if the above interaction between action preparation/execution and positive reward-signal detection and processing affects recruitment of physical resources regardless of whether the goal-priming words are consciously perceived, the affective-motivational effect should not differ between supraliminal and subliminal presentations.

As mentioned above, for explicit motor preparation, the assumption is that people consciously assess the goal state before developing conscious intentions or deliberate strategies to attain that state^27–29^. In contrast, previous studies have suggested that unconscious (implicit) goal-directed activity is produced by a different mechanism that uses the affective valence directly attached to representations of behavioural states to automatically direct effort toward attaining those states^18,30^. Thus, unconscious goal pursuit, which is triggered by a positive affect that is associated with the representation of the primed behavioural state, will emerge independently from conscious goal pursuit.

Here, we aimed to clarify this issue and determine how awareness of a motor goal can modulate the effect of affective motivational priming in a motor task by using the same paradigm and conceptual analysis as those in an earlier study^23^. We ensured conscious reward-goal priming by presenting the priming words for an extended duration, and then compared how supraliminal and subliminal reward-goal priming affect the motor and pupil-linked neuromodulatory systems. Our results demonstrated that both types of affective behavioural goal priming can influence these systems such that voluntary motor actions are more forceful, possibly via enhanced dopaminergic activity.

## Materials and Methods

### Participants

Thirty-six healthy Japanese right-handed individuals (evaluated using the Edinburgh Handedness Inventory^31^) participated (23 males, 13 females; mean age ± SD: 21.7 ± 3.2 years) in the study after providing both written and oral informed consent according to the Declaration of Helsinki (1991; p. 1194). We confirmed that pregnant women were not among our participants to avoid the unknown risks of TMS on an unborn foetus. The experimental procedures complied with relevant laws and institutional guidelines, and were approved by the Human Research Ethics Committee of the Faculty of Sport Sciences at Waseda University (approval number 2014–272).

### Stimuli and the task

**Figure 1:**
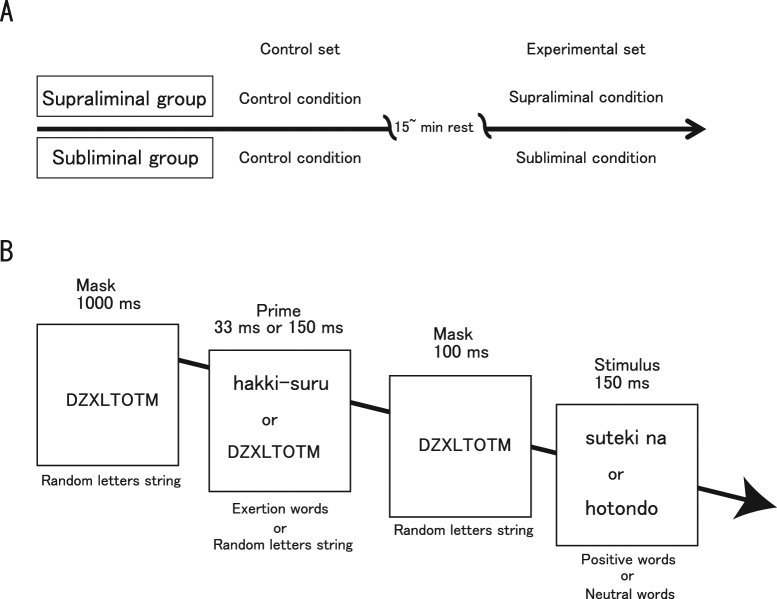
Experimental procedures. (**A**) The timeline of a trial for each condition and group. Participants were randomly assigned to one of two groups: supraliminal or subliminal. Each group participated in two (control and experimental) conditions, with a break of at least 15 min between each. Both supraliminal and subliminal groups completed the control condition first, followed by supraliminal or subliminal condition, respectively. (**B**) Priming procedure. In the subliminal condition, the subliminal exertion primes were always paired with supraliminal positive words. The supraliminal condition was identical, except that the initial presentations of exertion verbs and random letters were fully visible (supraliminal). Thus, the subliminal and supraliminal exertion primes were always paired with positive words. In the control condition, only random letter strings were used as primes, and these were paired with positive or neutral words. Thus, exertion primes were never displayed. The order of possible stimulus pairs was randomized within each condition. Exertion, positive, and neutral words were Japanese. Each trial in each condition began with a 1000-ms presentation of a random eight letter string (e.g., DZXLTOTM) as a forward mask. This was followed by the prime (33-ms duration in the subliminal condition and 150-ms duration in the supraliminal condition). A random letter string was again displayed for 100 ms as a backward mask, after which a consciously visible word was presented for 150 ms. Occasionally, a dot was presented for 33 ms (it was visible because of the absence of a backward mask), either above or below the neutral or positive word.

We opted for a between-participants design to optimize enhancement of the motor system (corticospinal excitability) via subliminal goal-priming with rewards^4,5^: the choice of the between-participants design prevents enhancement of corticospinal excitability. Participants were randomly assigned to one of two groups: supraliminal or subliminal. Each group (n = 18) received two (control and experimental) sets of tests, with a break of at least 15 min between sets. Both groups completed the control condition (random letter primes with subsequent “reward” words) before their respective experimental conditions. In the experimental condition, priming words related to physical exertion were paired with subsequently displayed “reward” words. The primes were invisible for the subliminal group and visible for the supraliminal group (Fig. 1A).

To investigate the excitability of the motor cortex, TMS was applied to the left primary motor cortex (M1) 1.5 s after the positive or neutral word (see “*Priming procedure*” below for details) disappeared. Thus, 50 motor-evoked potentials (MEPs) were obtained for each condition. We investigated the influence of conscious goal pursuit on the pupil-linked noradrenergic system by examining pupil dilation during each manipulation. To examine each condition’s effect on motor behaviour, after each priming condition, we asked participants to squeeze a handgrip-force apparatus three times with their right hand (5 s per squeeze), with a 6-s inter-squeeze interval.

### Testing of subliminal stimuli

To confirm that the subliminal primed words were not consciously perceived, we conducted a separate experiment in which different participants (32 males and 8 females; mean age: 22.05 ± 2.07 years) completed the subliminal and supraliminal conditions. Participants were asked to indicate whether they saw a word related to physical exertion. The post-masked subliminal exertion-related primes were attended, but not reported. Response accuracy was 50.15% ± 6.23%, indicating that their judgements did not differ from chance, and that they could not see the priming words.

### Priming procedure

To manipulate unconscious goal pursuit, we adopted an experimental procedure used in previous studies^4,23^ (Fig. 1B). Specifically, we used five Japanese verbs related to physical exertion (‘to exert’ [hakki-suru], ‘to struggle’ [funtou-suru], ‘to work hard’ [mogaku], ‘to energize’ [sei wo dasu], and ‘to strive’ [doryoku suru]) as motor goals, five positive adjectives (‘nice’ [suteki na], ‘great’ [subarashii], ‘fantastic’ [kibunsaikou no], ‘satisfactory’ [manzoku na], and ‘enjoyable’ [tanoshii]) as positive reward, and five neutral adverbs (‘almost’ [hotondo], ‘at least’ [sukunaku tomo], ‘finally’ [saigoteki ni], ‘nearly’ [hobo], and already [sude ni]) as non-reward words. For the subliminal group, for half of the 50 experimental trials, subliminal presentation of one of the five exertion verbs was followed by supraliminal presentation of one of the five positive adjectives. For the remaining 25 trials, subliminal presentation of a random letter string was followed by supraliminal presentation of one of the five neutral words. For the supraliminal group, the pattern was identical, except that the initial presentations of exertion verbs and random letters were fully visible. Thus, the exertion primes were always paired with positive words. In the control condition, only random letter strings were used as primes, and these were paired with positive words on 25 trials and with neutral words on 25 trials. Note that exertion words (subliminal or supraliminal) were never displayed in the control condition. In this way, the viewing of positive and neutral words was balanced at 25 trials apiece in all conditions. The 25 trials for each group of word pairs in each condition comprised exactly one presentation of each possible word pair (5 initial words × 5 subsequent words), the order of which was randomized.

Each trial in each condition began with a 1000-ms presentation of five different strings of eight pseudorandom letters (DZXLTOTM, YSTZBXTU, VCFTHYPC, CBEXGTVY, and ZTAWYDBH) as a forward mask. This was followed by a 33-ms presentation of the subliminal prime or a 150-ms presentation of the supraliminal prime, depending on the group. One randomly selected letter string among the original five was again displayed for 100 ms as a backward mask, after which a supraliminal word was presented for 150 ms. Occasionally, a dot was presented for 33 ms (visible because of the absence of a backward mask), either above or below the neutral or positive word. Participants were instructed to indicate whether they had seen a dot, which served to bring the post-masked subliminal primes to their attention. Trials were carried out every 3.5 s within each condition. We used a 60-Hz CRT screen to display the words, and the experimental procedure was created with software designed for psychological experiments (Inquisit 3 Desktop Edition, Millisecond Software, Seattle, WA, USA). All word stimuli were displayed in black on a white screen during priming, and immediately before the word presentation, the colour of the screen was momentarily white without any black words. Thus, pupil diameter transiently decreased because of the preceding large increase in luminance by the white screen with the maximum luminance. We are unable to completely eliminate the possibility that this transient change in luminance affected pupil diameter; however, this type of contamination should be small because this phenomenon was common across all participants.

### Handgrip force measurement and subjective perception of effort

Force was measured using a handgrip device (KFG-5-120-C*1-1*6, Kyowa Electronic Instruments, Tokyo, Japan). After viewing all 50 stimulus pairs, participants completed a squeeze task in which they were asked to squeeze the handgrip device with their right (dominant) hand when the word ‘squeeze’ appeared on the display, and to stop squeezing when the word disappeared. The squeeze instruction was displayed for 5 s, and was repeated three times with a 6-s inter-squeeze interval. After completing the squeeze task, participants were asked to rate how hard they had tried to squeeze the device on a category-ratio (CR-10) scale^32^. A rating of 1 signified ‘very weak’, and 10 signified ‘very, very strong’. Reaction time (time from the ‘squeeze’ instruction to the production of handgrip force), the rate of force increase (the first peak in force curve divided by the time of the first peak), and total effort (mean force over time) were quantified as motor actions (behaviours) from the force curve that was obtained based on paradigms used in previous research^4,23^.

### Transcranial magnetic stimulation (TMS)

In both control and experimental conditions, single-pulse TMS was administered via a stimulator (M200^2^, Magstim, Whitland, Dyfed, UK) using a coil with a double, figure-eight shaped winding (4150-00 Double 70 mm Alpha Coil, Magstim, Whitland, Dyfed, UK) with a maximum magnetic field strength of 1.55 T. The stimulation was delivered 1.5 s after the positive or neutral words disappeared in each trial. Each participant sat upright with their elbows bent in front of them and resting on their thighs. The TMS coil was positioned over the finger area of the left M1, which was determined as the area with the lowest resting motor threshold (rMT). That is, the area for which MEPs with peak-to-peak amplitudes greater than 50 µV were induced in the flexor carpi ulnaris (FCU) muscle in at least 5 out of 10 trials when participants were totally relaxed with their eyes closed^33,34^. Coil position was stabilized throughout the experiment with a coil stand made from multiple products (Manfrotto Distribution KK, Tokyo, Japan). The optimal scalp position of M1 was marked directly on the scalp with a black magic marker. Here, rMTs ranged from 50% to 85% of the maximum stimulator output, and stimulus intensity for each participant was set at 110% of their rMT.

During MEP recording, participants were asked to remain in a resting state. Surface electromyograms were obtained from the right FCU muscles with bipolar surface silver electrodes (bandpass, 15–10 kHz) using the tendon-belly method. For each condition, the peak-to-peak amplitude of the averaged MEP across 50 trials was calculated, the size of which reflects corticospinal excitability^35,36^. The background electromyogram (EMG) was calculated as the integral of the rectified EMG signal during the 100 ms before TMS.

### Pupil diameter measurement

We investigated the influence of conscious goal pursuit on the pupil-linked neuromodulatory system by examining modulations in pupil dilation. Pupil diameters were measured using a TalkEye Lite system (Takei Scientific Instruments Co., Ltd, Tokyo, Japan). The image around the pupil was obtained by a camera employing near-infrared light-emitting diodes (NIR LEDs) and a VGA (640 × 480) (DSP built in) Camera Module (NCM03-V, Nippon Chemi-Con Corporation, Tokyo, Japan). Banalization processing was performed on the image, and then pupil diameter was measured according to methods in Wang *et al*.^37^. Change in pupil size was estimated by considering the horizontal (*x*) and vertical (*y*) diameters in the experimental conditions relative to the control condition. Mean *x* and *y* diameters are expressed as a percentage of baseline values obtained from the control condition while participants viewed the 50 pairs of words.

### Skin potential level (SPL) measurement and range index

We also measured skin potential level (SPL). SPL is related to activation of postganglionic sympathetic fibres^38–40^, and is an indicator of general arousal. If goal pursuit is successfully induced unconsciously, SPL should not differ across the two experimental conditions. SPL was measured with Ag-AgCl skin electrodes (NS-111T and NS-115T, Nihon Kohden, Saitama, Japan), which were 7 mm in diameter and attached to the skin by 10-mm-diameter electrode collars (H261, Nihon Kohden). The face of each electrode was coated with a conductive electrolyte. Electrodes were tested in saline before experimental sessions to ensure a low and stable bias potential. The active electrode was placed on the thenar eminence of the left palm, which had been cleaned with ethanol. The inactive reference electrode was placed over the inner surface of the left forearm. The reference site was cleaned and abraded lightly with an electrode gel (YZ-0019, Nihon Kohden) at the beginning of each experimental session. SPL was amplified using a DC pre-amplifier and recorded onto a personal computer (Panasonic CF-R4, Tokyo, Japan).

The range index (*y*) of the SPL that corresponded to the original value (*x*) is defined by *y* = 100 × (*x* − *m*1)/(*M* − *m*2) (%), where *x* and *m*1 are the most negative and the least negative SPL values, respectively, that were obtained while viewing the 50 pairs of words in the experimental (supraliminal and subliminal) and control conditions, and where *M* and *m*2 are the most negative and the least negative SPL values obtained during a 5-min closed-eyed resting state before the tasks^40^.

### Statistical analysis

Motor behaviour, MEP, pupil size, and SPL data were analysed using repeated-measures two-way analyses of variance (ANOVAs) with a between-participant factor of Group (supraliminal or subliminal) and a within-participant factor of Condition (control or experimental. Greenhouse–Geisser corrections were applied when appropriate to adjust for non-sphericity, changing the degrees of freedom using a correction coefficient. A significance threshold of *p* < 0.05 was chosen for all tests. We also performed a Bayesian Repeated Measures ANOVA on the data (repeated-measures factor: Condition; between-participants factor: Group) using JASP (https://jasp-stats.org/) to quantify how the observed data supported evidence in favour of the null hypothesis (i.e., the absence of difference between supraliminal and subliminal groups).

### Data Availability

All data generated or analysed for this study are included in this published article.

## Results

### Motor action

#### Effort

**Figure 2:**
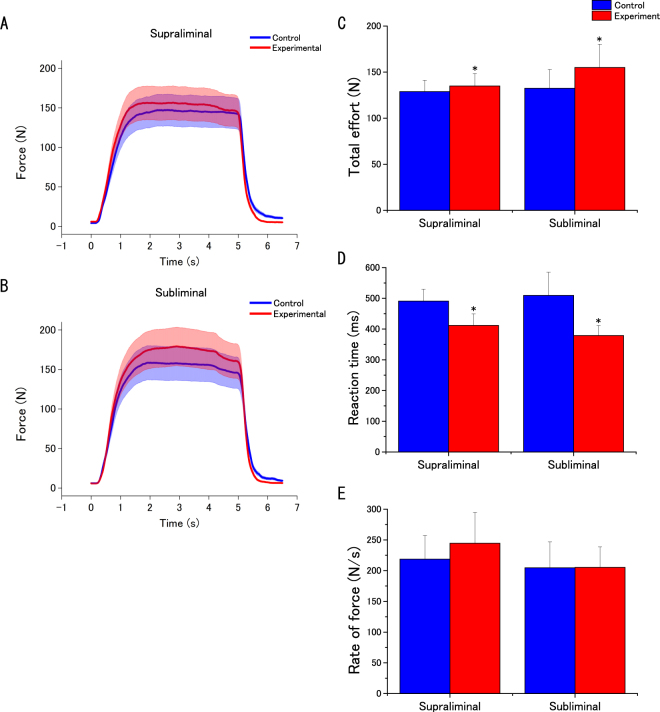
Effect of unconscious goal pursuit on motor action. (**A**,**B**) Typical recordings of handgrip force in each experimental (supraliminal or subliminal) condition. (**C**,**D**,**E**) Total effort (**C)**, reaction time (**D**), and rate of force (**E**) for the two conditions. Data are expressed as mean ± SEM. *Experimental vs. control condition *p* < 0.05.

We found that total effort was enhanced in both the supraliminal and subliminal experimental conditions. A two-way ANOVA revealed a significant main effect of Condition (control vs. experimental for supraliminal and subliminal groups) on total effort (*F*_(1,34)_ = 7.464; MSE = 494.32; *p* = 0.010; effect size: partial *η*^2^ = 0.1800), but no significant effect of Group (supraliminal vs. subliminal) (*F*_(1,34)_ = 0.223; MSE = 11277.66; *p* = 0.640; effect size: partial *η*^2^ = 0.007), or any interaction between Condition and Group (*F*_(1,34)_ = 2.53; MSE = 494.32; *p* = 0.121; effect size: partial *η*^2^ = 0.069) (Fig. 2A–C; Table 1).

**Table 1:** Motor action responses for the two conditions.

	Reaction time (ms)		Rate of force (N/s)		Total effort (N)	
	Control set	Experimental set	Control set	Experimental set	Control set	Experimental set
Supraliminal	491.2 ± 37.5	412.0 ± 36.5*	218.4 ± 37.3	244.7 ± 48.7	128.8 ± 11.8	134.8 ± 13.1
Subliminal	509.6 ± 64.6	378.9 ± 28.2*	204.6 ± 36.2	205.4 ± 28.6	132.3 ± 17.5	155.0 ± 21.6*

Values indicate means ± SEM.

^*^Significant differences at p < 0.05 vs. values from the control condition.

#### Reaction time

We found that reaction times tended to decrease in both groups in the experimental tasks. A two-way ANOVA revealed a significant main effect of Condition (*F*_(1,34)_ = 5.209; MSE = 38064.86; *p* = 0.029; effect size: partial *η*^2^ = 0.133), but no significant effect of Group (*F*_(1,34)_ = 0.022; MSE = 43883.39; *p* = 0.883; effect size: partial *η*^2^ = 0.001), or any interaction between Condition and Group (*F*_(1,34)_ = 0.314; MSE = 38064.86; *p* = 0.579; effect size: partial *η*^2^ = 0.009) (Fig. 2A,B,D; Table 1).

#### Rate of forc

No significant changes were observed in either group relative to the control condition. A two-way ANOVA revealed no significant main effects or any interaction between factors (Condition: *F*_(1,34)_ = 1.539; MSE = 2098.21; *p* = 0.223; effect size: partial *η*^2^ = 0.043; Group: *F*_(1,34)_ = 0.227; MSE = 56391.9; *p* = 0.637; effect size: partial *η*^2^ = 0.007; Interaction: *F*_(1,34)_ = 1.367; MSE = 2098.21; *p* = 0.250; effect size: partial *η*^2^ = 0.039) (Fig. 2A,B,E; Table 1).

#### Subjective effort

Although subjective effort increased in both groups relative to the control condition (subliminal: control baseline, 5.94 ± 0.42; subliminal condition, 6.27 ± 0.41; supraliminal: control baseline, 5.83 ± 0.38; supraliminal condition, 6.33 ± 0.43), the two-way ANOVA revealed that the effect of Condition just missed significance (*F*_(1,34)_ = 3.899; MSE = 0.801; *p* = 0.056; effect size: partial *η*^2^ = 0.103). The analysis also showed no significant main effect of Group (*F*_(1,34)_ = 0.003; MSE = 5.347; *p* = 0.960; effect size: partial *η*^2^ = 0.000076) or a significant interaction (*F*_(1,34)_ = 0.156; MSE = 0.801; *p* = 0.695; effect size: partial *η*^2^ = 0.005).

### Motor evoked potential (MEP)

**Figure 3:**
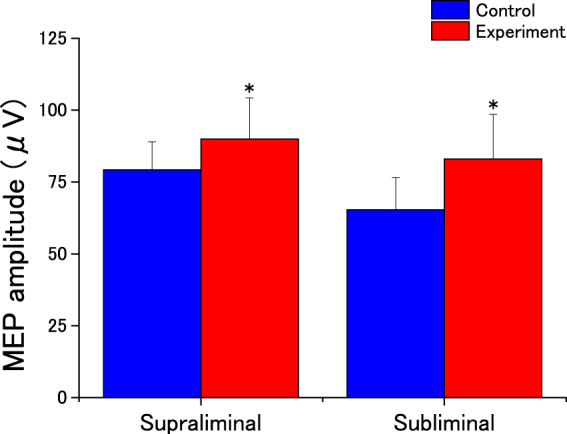
Effects of unconscious goal pursuit on motor-evoked potential (MEP) amplitude. Amplitudes of MEPs of the flexor carpi ulnaris for the two experimental conditions. Data are expressed as mean ± SEM. Asterisks indicate statistically significant differences compared with control condition (*p < 0.05).

We found that compared with the control condition, MEP amplitude was enhanced after both supraliminal and subliminal priming (Fig. 3). The two-way ANOVA revealed that this effect of Condition was indeed significant (*F*_(1,34)_ = 7.864; MSE = 463.24; *p* = 0.008; effect size: partial *η*^2^ = 0.188). Additionally, it showed no significant effect of Group (*F*_(1,34)_ = 0.373; MSE = 5214.14; *p* = 0.545; effect size: partial *η*^2^ = 0.011) or any interaction between Condition and Group (*F*_(1,34)_ = 0.475; MSE = 463.24; *p* = 0.495; effect size: partial *η*^2^ = 0.014). These results show that priming with goal-related exertion words acted to excite corticospinal activity in both groups.

We also observed that background EMG decreased in both groups. Analysis revealed a significant main effect of Condition (*F*_(1,34)_ = 12.542; MSE = 7224.52; *p* = 0.001; effect size: partial *η*^2^ = 0.269), but no significant main effect of Group (*F*_(1,34)_ = 0.148; MSE = 106651.47; *p* = 0.703; effect size: partial *η*^2^ = 0.004) and no significant interaction between Condition and Group (*F*_(1,34)_ = 0.012; MSE = 7224.52; *p* = 0.912; effect size: partial *η*^2^ = 0.00036).

The behavioural and MEP results showed that behavioural goal priming with positive (motivational) words enhanced corticospinal excitability despite the lack of conscious awareness, as evidenced by the increased total force and MEP amplitude, the lower reaction times in the experimental conditions, and the lack of a main effect for Group in any test. Thus, both subliminal and supraliminal primed behavioural goals lead to faster and more forceful voluntary motor action.

### Pupil diameter and skin potential level (SPL)

**Figure 4:**
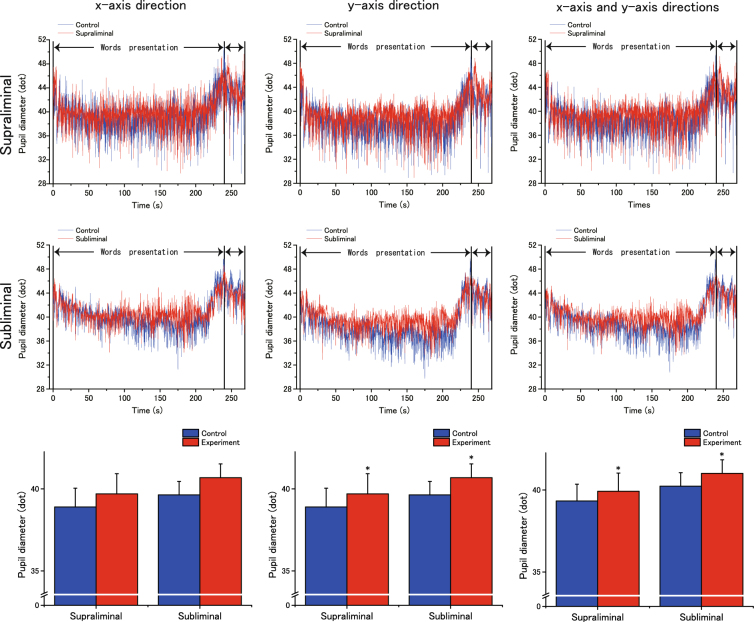
The effects of unconscious goal pursuit on pupil size over time. *Top and middle rows*. Horizontal (left), vertical (middle), and horizontal + vertical (right) pupil diameter (dots) starting at the onset of word presentation in the priming procedure and lasting until the end of the handgrip task. Diameter is expressed as the mean for each experimental (supraliminal or subliminal) group. The data were low-pass filtered with a cut-off frequency of 1 Hz using a fourth-order Butterworth filter. The bidirectional arrows (↔) after the word presentation period indicate the period of the handgrip task. *Bottom row*. Pupil-size data are expressed as mean ± SEM. *p < 0.05, vs. control condition.

Figure 4 shows the time-course of pupil-diameter measures from the onset of word presentation to the third disappearance of the word ‘squeeze’. In both groups, we found that pupil size increased in the vertical (*y*) and horizontal and vertical (*x* and *y*) directions during the priming procedure. Analysis revealed that this effect of Condition was significant (vertical: *F*_(1,34)_ = 12.77; MSE = 2.094; *p* = 0.001; effect size: partial *η*^2^ = 0.273; horizontal and vertical: *F*_(1,34)_ = 9.640; MSE = 1.359; *p* = 0.004; effect size: partial *η*^2^ = 0.221). It also showed no significant effect of Group (vertical: *F*_(1,34)_ = 0.082; MSE = 2.094; *p* = 0.777; effect size: partial *η*^2^ = 0.002; horizontal and vertical: *F*_(1,34)_ = 0.002; MSE = 1.359; *p* = 0.965; effect size: partial *η*^2^ = 0.00005) or any interaction between Condition and Group (vertical: *F*_(1,34)_ = 0.632; MSE = 37.95; *p* = 0.432; effect size: partial *η*^2^ = 0.018; horizontal and vertical: *F*_(1,34)_ = 0.714; MSE = 35.75; *p* = 0.404; effect size: partial *η*^2^ = 0.021). Additionally, pupil size tended to increase horizontally in both groups (Fig. 4), but the increase was not statistically significant (Condition: *F*_(1,34)_ = 3.35; MSE = 1.276; *p* = 0.076; effect size: partial *η*^2^ = 0.009). As with other tests, no main effect of Group (*F*_(1,34)_ = 0.680; MSE = 39.86; *p* = 0.415; effect size: partial *η*^2^ = 0.002) or any interaction between Condition and Group (*F*_(1,34)_ = 0.075; MSE = 1.276; *p* = 0.785; effect size: partial *η*^2^ = 0.002) was observed.

SPL on the palmar surface did not appear to change in either the supraliminal (control: 183.8 ± 37.2%; experimental: 186.9 ± 51.2%) or subliminal (131.8 ± 13.4%, 136.9 ± 22.1%) groups. Analysis confirmed this by indicating no main effects or any interaction (Condition: *F*_(1,34)_ = 0.025; MSE = 11882.06; *p* = 0.875; effect size: partial *η*^2^ = 0.001; Group: *F*_(1,34)_ = 1.564; MSE = 29974.87; *p* = 0.220; effect size: partial *η*^2^ = 0.044; Interaction: *F*_(1,34)_ = 0.002; MSE = 11882.00; *p* = 0.967; effect size: partial *η*^2^ = 0.00005). This indicates that the experiment did not induce any significant changes in the level of general arousal.

### Results of bayesian testing

The classical statistical testing described above demonstrated there was no significant interaction between condition and group. However, this result cannot be interpreted as the evidence of supporting the absence of interaction. Thus, in order to examine more explicitly how the absence of interaction was supported by the observed data, we also performed Bayesian testing^41,42^. Tables 2–10 summarized the results of Bayesian testing. As for the effort (Table 2), the BF_01_ for Condition + Group, and that for Condition + Group + Condition*Group are 0.398 and 0.453, respectively. The addition of interaction increased the BF_01_ by a factor of 1.14 (=0.453/0.398) and the similar testing results were obtained for other variables (Tables 3–9). The factors by the inclusion of interaction greater than 1 (1.14~3.22), indicating that the data supported more likely the absence of interaction. However, it should be noted that those values are classified as anecdotal evidence^43^. Therefore, it is clear that both subliminal and supraliminal goal-priming with positive words increased the variables, while we should be cautious about the interpretation that the degree of increase was identical between the two conditions.

**Table 2:** Bayesian repeated-measures ANOVA results. All models include subject.

Effort					
Model Comparison	P(M)	P(M|data)	BF_M_	BF_01_	error %
Models					
Null model (incl.subject)	0.200	0.097	0.432	1.000	
Condition	0.200	0.379	2.490	0.254	1.824
Group	0.200	0.065	0.251	1.653	2.522
Condition+ Group	0.200	0.244	1.298	0.398	2.903
Condition+ Group+ Condition*Group	0.200	0.215	1.095	0.453	3.347

**Table 3:** Bayesian repeated-measures ANOVA results. All models include subject.

Reaction time					
Model Comparison	P(M)	P(M|data)	BF_M_	BF_01_	error %
Models					
Null model (incl.subject)	0.200	0.169	0.816	1.000	
Condition	0.200	0.546	4.818	0.310	0.705
Group	0.200	0.052	0.211	3.234	5.300
Condition+ Group	0.200	0.170	0.822	0.993	4.229
Condition+ Group+ Condition*Group	0.200	0.061	0.262	2.769	1.961

**Table 4:** Bayesian repeated-measures ANOVA results. All models include subject.

Rate of force					
Model Comparison	P(M)	P(M|data)	BF_M_	BF_01_	error %
Models					
Null model (incl.subject)	0.200	0.384	2.491	1.000	
Condition	0.200	0.171	0.825	2.244	0.792
Group	0.200	0.269	1.401	1.480	2.711
Condition+ Group	0.200	0.120	0.548	3.187	3.225
Condition+ Group+ Condition*Group	0.200	0.065	0.280	5.885	3.751

**Table 5:** Bayesian repeated-measures ANOVA results. All models include subject.

Subjective effort					
Model Comparison	P(M)	P(M|data)	BF_M_	BF_01_	error %
Models					
Null model (incl.subject)	0.200	0.293	1.657	1.000	
Condition	0.200	0.347	2.124	0.845	0.678
Group	0.200	0.134	0.621	2.180	1.846
Condition+ Group	0.200	0.168	0.806	1.747	2.188
Condition+ Group+ Condition*Group	0.200	0.058	0.247	5.038	2.626

**Table 6:** Bayesian repeated-measures ANOVA results. All models include subject.

MEP					
Model Comparison	P(M)	P(M|data)	BF_M_	BF_01_	error %
Models					
Null model (incl.subject)	0.200	0.088	0.387	1.000	
Condition	0.200	0.460	3.405	0.192	0.790
Group	0.200	0.051	0.214	1.734	2.037
Condition+ Group	0.200	0.289	1.630	0.305	2.349
Condition+ Group+ Condition*Group	0.200	0.112	0.503	0.790	2.836

**Table 7:** Bayesian repeated-measures ANOVA results. All models include subject.

Pupil size in the x direction					
Model Comparison	P(M)	P(M|data)	BF_M_	BF_01_	error %
Models					
Null model (incl.subject)	0.200	0.269	1.470	1.000	
Condition	0.200	0.261	1.409	1.031	1.170
Group	0.200	0.213	1.082	1.262	8.158
Condition+ Group	0.200	0.193	0.957	1.392	2.072
Condition+ Group+ Condition*Group	0.200	0.065	0.277	4.146	3.003

**Table 8:** Bayesian repeated-measures ANOVA results. All models include subject.

Pupil size in the y direction					
Model Comparison	P(M)	P(M|data)	BF_M_	BF_01_	error %
Models					
Null model (incl.subject)	0.200	0.017	0.068	1.000	
Condition	0.200	0.510	4.157	0.033	1.357
Group	0.200	0.011	0.045	1.608	0.654
Condition+ Group	0.200	0.349	2.140	0.048	1.110
Condition+ Group+ Condition*Group	0.200	0.114	0.515	0.148	2.279

**Table 9:** Bayesian repeated-measures ANOVA results. All models include subject.

Pupil size in the x and y directions					
Model Comparison	P(M)	P(M|data)	BF_M_	BF_01_	error %
Models					
Null model (incl.subject)	0.200	0.045	0.187	1.000	
Condition	0.200	0.472	3.583	0.094	1.904
Group	0.200	0.032	0.134	1.378	1.336
Condition+ Group	0.200	0.341	2.071	0.131	2.875
Condition+ Group+ Condition*Group	0.200	0.109	0.491	0.408	2.549

**Table 10:** Bayesian repeated-measures ANOVA results. All models include subject.

SPL					
Model Comparison	P(M)	P(M|data)	BF_M_	BF_01_	error %
Models					
Null model (incl.subject)	0.200	0.483	3.733	1.000	
Condition	0.200	0.116	0.526	4.152	1.269
Group	0.200	0.304	1.747	1.588	0.833
Condition+ Group	0.200	0.074	0.320	6.520	2.389
Condition+ Group+ Condition*Group	0.200	0.023	0.094	21.043	2.050

## Discussion

Here we showed that pupil diameter and MEP increased significantly when participants were presented either subliminal or supraliminal goal-priming words that were followed by explicit positive reward words. Similarly, the submaximal force level measured after the priming procedure was also enhanced. These changes occurred without any detectable changes in general arousal state, as demonstrated by the SPL results. Our findings indicate that the motor and pupil-linked neuromodulatory systems were more excitable regardless of whether the goal-priming words were visible or not, and that this led to more forceful voluntary motor action.

The participants in the supraliminal group saw all the primes and were able to produce more forceful voluntary motor action through the enhancement of the corticospinal pathway. They may have consciously assessed the primes and adopted the desired goal state; thus, the activation of the motor system including the PMC via motor-goal representation could have involved in the enhancement of the corticospinal pathway^25,26^. Alternatively, the mental representations of goals might have been activated without an act of conscious will, guiding a participant’s motor goal-relevant cognition, affect, and motor behaviour from the activated point onward^30,44,45^. Thus, the goal-priming effect on task performance might have resulted from unconscious activation of the goal to perform well rather than the conscious perception of the goal. Indeed, the debriefing after the experimental task indicated that participants in the supraliminal group had been aware of the exertion words; however, none realized the true nature of the study, particularly the idea of affective shaping or the link between affective shaping and subsequent motor performance. Thus, they were unaware of any activated motor goals or any causal linkage between the goal-priming words and the positive reward words during reward goal-priming manipulation. This means that the motor-goal pursuit was triggered unconsciously. Meanwhile, they did not experience any subjective difference in effort immediately after the motor action as participants in the subliminal group did. This suggests that the enhancing effect of supraliminal goal priming with rewards in physical resources was automatic. At the same time, we must keep in mind that memory-based self-report (such as the CR-10 scale) is not the most sensitive or powerful measure of actual awareness during past actions^30^. Therefore, based on the proposed mechanism for unconscious goal pursuit^24^, an association of a motor-goal representation with positive affect can similarly arise in the background of consciousness and act as a reward signal, inducing unconscious motivation for the motor goal, regardless of whether the goal is consciously perceived or not. This leads motor action to be enhanced in the same way as when conscious perception of the goal barely exists.

Delivering several single TMS pulses repeatedly over M1 has been shown to induce cumulative changes in neural activity during stimulation, which serves to increase motor cortical excitability within the same block of stimulation^46^. This possibility was never completely excluded in our study; however, we were convinced that such a cumulative effect on corticospinal excitability would not be able to conceal the increase in corticospinal excitability that was induced by rewarding physical exertion primes in our study. This was because the trend analyses for the cumulative change have been shown to have a perfect linear trend (R2 = 1) for inter-block levels and a nearly linear trend for intra-block levels (R2 = 0.97)^46^.

Pupillometry at steady luminance has long been utilized as an indirect measure of brain states associated with cognition that involves mental effort^15–17^. Pupil dilation is directly and/or indirectly modulated by the release of noradrenaline from the locus coeruleus (LC) that acts on noradrenergic α2-receptors and is proposed to be causally responsible for pupil dilation, and is thus widely used as an indirect marker of LC activity^7–9^. LC activation does not influence the neurons in the ventral tegmental area (VTA) that project to the nucleus accumbens, which is implicated in dopamine release. However, as neurons in the prefrontal cortex (PFC) project indirectly to the VTA, LC activation regulates reciprocal connections between the noradrenergic and dopaminergic systems, and their reciprocal associations in the PFC^6^. These two biochemically analogous catecholaminergic neuromodulatory systems are functionally and anatomically tightly associated.

Therefore, potentiated dopaminergic activity might enhance activity within the pupil-linked noradrenergic system, which in turn might boost the gain of neuronal interactions in the cortex^8,47^ and facilitate goal pursuit outside of conscious awareness via simultaneous action preparation and the detection of positive reward signals. It might also potentiate the reciprocal actions between the noradrenergic and dopaminergic systems and their reciprocal associations within the PFC. In fact, the role of the noradrenergic system in motivation of goal pursuit has been proposed to complement that of the dopaminergic system^48,49^. The dopaminergic system is involved in value-based decision making, whereas the noradrenergic system is involved in energizing behaviour and enhancing efforts to face challenges. Taken together, the current results also suggest that unconscious goal pursuit might enhance the noradrenergic system and be relevant to more potently motivated voluntary motor behaviour, perhaps via potentiation of dopaminergic system activity. Additionally, we must keep in mind that because of the length of the presented word—and other factors—the difference in screen luminance might have had some effect on pupil diameter. However, we believe that even if this contamination were real, it would not cancel out the observed pupil dilation. This is because the observed pupil dilation in both the supraliminal and subliminal groups was accompanied by enhanced corticospinal excitability. This is consistent with our previous study, which showed how the affective motivational effect on the motor system is related to both the reward-associated dopaminergic system and the pupil-linked neuromodulatory system^5^.

We note that the potentiation of the pupil-linked neuromodulatory system and the more forceful voluntary motor actions occurred regardless of whether the goal-priming words were fully visible or not. This indicates that conscious awareness of behavioural goal-priming is not a necessary condition for goal pursuit processes to recruit resources for motor action. This in turn suggests that the effect of implicit motivation, which is induced by an association between physical exertion and positive affect, underlies motor-goal priming and acts as a reward signal.
